# Drought-induced trans-generational tradeoff between stress tolerance and defence: consequences for range limits?

**DOI:** 10.1093/aobpla/plt038

**Published:** 2013-08-30

**Authors:** Jacob D. Alsdurf, Tayler J. Ripley, Steven L. Matzner, David H. Siemens

**Affiliations:** 1 Department of Biology, Black Hills State University, Spearfish, SD, USA; 2 Integrative Genomics Program, Black Hills State University, Spearfish, SD, USA; 3 Department of Biology, Augustana College, Sioux Falls, SD, USA

**Keywords:** *Boechera stricta*, chemical defence, drought tolerance, epigenetic, glucosinolate, range limit, tradeoff

## Abstract

Areas just across species range boundaries are often stressful, but even with ample genetic variation within and among range-margin populations, adaptation towards stress tolerance across range boundaries often does not occur. Adaptive trans-generational plasticity should allow organisms to circumvent these problems for temporary range expansion; however, range boundaries often persist. To investigate this dilemma, we drought stressed a parent generation of *Boechera stricta* (A.Gray) A. Löve & D. Löve, a perennial wild relative of *Arabidopsis*, representing genetic variation within and among several low-elevation range margin populations. *Boechera stricta* is restricted to higher, moister elevations in temperate regions where generalist herbivores are often less common. Previous reports indicate a negative genetic correlation (genetic tradeoff) between chemical defence allocation and abiotic stress tolerance that may prevent the simultaneous evolution of defence and drought tolerance that would be needed for range expansion. In growth chamber experiments, the genetic tradeoff became undetectable among offspring sib-families whose parents had been drought treated, suggesting that the stress-induced trans-generational plasticity may circumvent the genetic tradeoff and thus enable range expansion. However, the trans-generational effects also included a conflict between plastic responses (environmental tradeoff); offspring whose parents were drought treated were more drought tolerant, but had lower levels of glucosinolate toxins that function in defence against generalist herbivores. We suggest that either the genetic or environmental tradeoff between defence allocation and stress tolerance has the potential to contribute to range limit development in upland mustards.

## Abstract (precis)

In the study of geographic range boundary development, the focus has been on leading rather than on trailing edge dynamics. This is an important caveat as trailing edge dynamics will be critical for an understanding population level persistence. Our study begins to fill this knowledge gap and extends the conceptual framework of the field by focusing on trans-generational environmental effects. We found that while these effects may overcome some constraints on stress tolerance evolution and range expansion, other constraints may be created to limit range.

## Introduction

A central question in evolutionary ecology is what are the factors and processes that contribute to the development of species range limits (Parmesan *et al*. 2005; Gaston 2009; Sexton *et al*. 2009; Wiens 2011; Anderson *et al*. 2012). Most transplant experiments show that areas just across geographic range boundaries are stressful (Sexton *et al*. 2009 for review), suggesting that organisms are often at their physiological limits at range edges. As such, adaptation for stress tolerance would be required to occupy these stressful environments. However, since range boundaries exist, several genetic constraints apparently prevent this adaptation. Here, we asked why trans-generational plasticity does not allow for temporary range expansion by overcoming any genetic constraints that may limit range.

Trans-generational phenotypic plasticity is the inheritance of environmentally induced effects without corresponding genetic (DNA sequence) changes, where the environmental cues are in a parent generation, but where the effects of interest are in offspring or subsequent generations (Herman and Sultan 2011; Holeski *et al*. 2012). Thus, inheritance of temporary phenotypic changes should occur in the absence of the genetic changes required for more permanent adaptation across the range boundaries.

We studied the effects of drought-induced trans-generational plasticity on factors affecting the range limit dynamics in *Boechera stricta* (A.Gray) A. Löve & D. Löve (Brassicaceae). *Boechera stricta* is a genetically diverse, predominantly self-fertilizing perennial and a close relative of *Arabidopsis thaliana* that ranges across the mountainous regions of western North America (Song *et al*. 2006,  2009). Our seed collection sites were mainly in a relatively low-elevation region for *B. stricta* located in the northern Black Hills of South Dakota. The Black Hills is an isolated, low-elevation mountain range at the eastern edge of the geographic range of *B. stricta* (Lee and Mitchell-Olds 2011; Siemens and Haugen 2013). In general, species commonly face both abiotic and biotic stressors at range boundaries, especially at low elevations (Ettinger *et al*. 2011), yet the combination and interaction of these factors in the context of range limit dynamics have rarely been studied (Sexton *et al*. 2009, but see Siemens *et al*. 2009). Just across the low-elevation range boundaries, *B. stricta* faces several correlated abiotic and biotic stress gradients, including decreased water availability and increased herbivory by generalist insect herbivores. These factors reduced plant size and lowered survivorship in transplant experiments across the range boundaries (Siemens *et al*. 2009, 2012; Siemens and Haugen 2013). Thus, one would predict, at a minimum, the evolution of increases in both drought stress tolerance and resistance to herbivory for local range expansion or to survive climate change in these lower elevation habitats that may become even drier. Chemical defence allocation, however, might constrain the evolution of abiotic stress tolerance because of selection acting on antagonistic signalling pathways (Siemens *et al*. 2009, 2012; Siemens and Haugen 2013). Here, we examined the effect of drought-induced trans-generational plasticity on this genetic tradeoff, and we determined whether there were any conflicts among drought-induced trans-generational plastic responses (environmental tradeoff) that might also contribute to range limit development.

## Methods

Here, we describe three experiments—the first is the ‘Parent generation’ experiment, where we drought stressed plants and collected their seeds (offspring) to use in two ‘Offspring generation’ experiments. In the parent generation, we also examined the fitness effects of drought and checked for the predicted genetic tradeoff between drought tolerance and defence allocation. In the first offspring generation experiment, we determined (i) the effect of parental drought treatments on the predicted genetic tradeoff between drought tolerance and chemical defence allocation and (ii) whether the parental drought treatments induced a tradeoff in the offspring between drought tolerance and defence allocation (environmental tradeoff). In the second offspring generation experiment, we improved the experimental design to more rigorously test for the drought-induced trans-generational environmental tradeoff and measured drought tolerance in different ways to also help verify the results.

### Parent generation

#### Parent drought treatments

**Figure 1.**
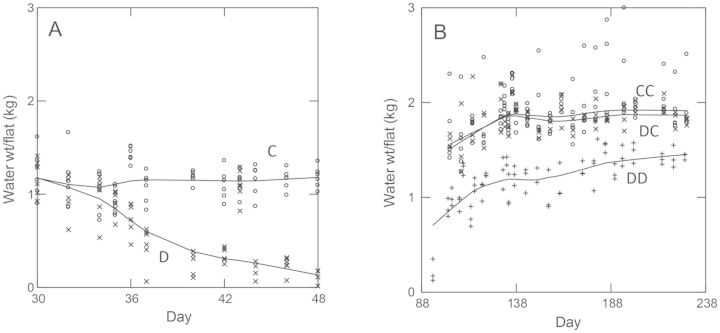
Watering treatments (C = Control, D = Drought) during the basal rosette stage (A), and during reproduction (i.e. bolting) (B), as measured by water weight per flat during the parent generation. Within a flat, water was distributed evenly among pots. Drought treatments began on Day 30 post planting. All flats were weighed before each watering. A cold treatment to induce flowering was administered to all flats between Days 48 and 88 post planting. Control flats were watered every other day to maintain the weights shown, while drought-treated flats were not watered until Day 48, and then periodically after cold treatments to maintain the weights shown. All of the control flats were continued as controls during reproduction (CCs), but half of the original drought-treated flats were treated as controls during reproduction (DCs), while the other drought-treated flats continued to be drought treated (DDs).

The parent generation was grown under three drought treatments: (i) control—CC, (ii) drought treated only during the basal rosette stage—DC and (iii) drought treated throughout the life cycle, i.e. during the basal rosette stage and during bolting and seed production—DD. Drought only during the rosette stage was an attempt to minimize any direct effects of drought on seed provisioning (i.e. carbohydrates, lipids, proteins and mineral nutrients). This created three parental drought-treatment groups (CC, DC and DD, respectively—see also the explanation given in Fig. 1), the trans-generational effects of which were studied in the offspring.

#### Genetic variation

We used seeds collected in the field from 64 plants (full-sib families, hereafter sib-families) representing the genetic variation within and among five relatively low-elevation sites in the northern Black Hills, SD, USA. Full-sib families were used because for self-fertilizing species like *B. stricta*, natural selection acts on the total genetic variation (additive and non-additive) and not exclusively on the additive genetic variation (Conner and Hartl 2004, p. 108).

#### Planting design

One seed from each sib-family (plant) collected from the field was randomized into 12 flats for a total sample size of 384 (1 plant/sib-family × 32 sib-families/flat × 2 sets of sib-families × 6 flats/set = 384). A flat was a tray that held 32 pots. The five populations were equally represented in the two sets of families. Planting flats contained 0.2-L pots filled with a soil mixture of two-thirds Premier ProMix BX and one-third sand, and 45.78 mg of 7:40:6 NPK MagAmp^®^ time release fertilizer. Flats were placed in a Percival growth chamber with a 16/8 h day/night photoperiod, 24 °/19 °C D/N temperature, under 12 100-watt incandescent and 28 WHO 1500 ma light bulbs.

#### Induction of flowering

On Day 48 post planting, flowering was induced with constant cold (to 4 °C) and low light (one fluorescent light bank) treatments to all flats. At this time, all flats were watered to control levels and fertilized with 1.4-g/L 20:20:20 NPK plus micronutrient (Peters) fertilizer. On Day 88, drought treatments were resumed, except that half of the drought-treated flats were assigned to control watering levels (Fig. 1).

#### Fitness effects

To determine the effects of the watering treatments on the parent generation, in addition to monitoring plant size and growth, we recorded flowering time, reproductive output, seed size and mass. Flowering time is an important component of reproductive isolation and would reduce gene flow to colonizing populations across the range boundary. Flowering time was recorded as the day of first flower. We conducted a census to determine the reproductive output on these indeterminate flowering plants (continuous flower production in the laboratory) by recording the number of fruits and any flowers and flower buds for each plant when plants were past peak flowering and producing fruits. After collecting mature seeds, we determined the average seed size for each plant by recording the length and width of three seeds using a light microscope. To determine whether the seed size correlated with seed mass, we weighed 100 seeds from each of the 12 sib-families representing the range of seed sizes observed across all of the flats.

#### Experimental design and statistical analysis

The design of the parental drought-treatment experiment was split-plot (Snedecor and Cochran 1967); drought treatments varying at the whole-flat level and population varying within flats. For statistical analysis, we used the following general linear-mixed model ANCOVA in SYSTAT 13:
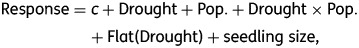

where Response is the response variable (rosette size, flowering time, reproduction or seed size), which were transformed as needed to satisfy assumptions of normality; *c* is the constant (*y* intercept); Drought denotes the watering treatments (Control or Drought), a fixed effect; Pop indicates the five different collecting sites, a random effect; ‘Flat(Drought)’, flat nested within the watering treatments, denotes the unmeasured random variation among flats not explained by the drought treatments and seedling size controlled for any slight variation in germination time, seed size and correlated maternal effects from the field. In preliminary analyses, we did not find the Pop-by-Flat(Drought) interaction to be important (*P* > 0.05); therefore, to simplify, we did not include this interaction in analyses. Consequently, the *F*-ratio for Drought was calculated using the mean square error (MSE) from Flat(Drought) in the denominator, whereas *F*-ratios for all other factors were calculated over the MSE_error_ (Zar 1996; Montgomery 1997). We used population instead of sib-family in this analysis, because all populations, but not all families, were represented within each flat.

#### Genetic tradeoff detection

To determine whether there was a genetic correlation between glucosinolate (GS) production and drought tolerance in the parent generation, we conducted regression analysis on the means of sib-families, controlling for random unmeasured variation among flats and for seedling size. That is, we first computed means of residuals from an analysis that only included Flat(Drought) and seedling size terms in the model. For each sib-family, tolerance to drought was measured as the root-to-shoot ratio and the slope of the reaction norm (Simms 2000) of mean performance (plant size) across drought treatments. An increased root-to-shoot ratio is a well-known drought tolerance response (Wilkinson and Davies 2002).

Previous studies (Siemens *et al*. 2009, 2012; Siemens and Haugen 2013) detected a genetic tradeoff between abiotic stress tolerance and basal GS levels. Thus, we measured basal GS values (μmol/mg) for each sib-family in the parent generation under control watering conditions. Glucosinolates are the notable defence metabolites of the Brassicaceae, which can have negative effects on a number of pests, including pathogens, generalist insect herbivores and interspecific neighbouring plants (Siemens *et al*. 2002; Lankau 2008; Schlaeppi *et al*. 2008; Bednarek *et al*. 2009).

#### Glucosinolate extraction and measurement

Glucosinolates were extracted in methanol, isolated on Sephadex ion-exchange columns and measured by high-performance liquid chromatography (HPLC) (Prestera *et al*. 1996; Brown *et al*. 2003). Briefly, half of each freeze-dried shoot was weighed, ground and extracted in 1.2 mL of methanol. We then added 500 µL of 1 mM aqueous allylglucosinolate (from Sigma) as an internal standard. To precipitate proteins, we added 50 µL of 0.6 M aqueous lead–barium acetate and centrifuged for 10 min at 3750 rpm. For further separation, the supernatant was added to a 0.6-mL DEAE A-25 Sephadex ion-exchange column, to which charged GSs bind by their sulfate group. To remove unwanted compounds that did not bind to Sephadex, the columns were rinsed twice with 1 mL of 67 % aqueous methanol and then once with 1 mL of distilled water. Columns were then rinsed with 0.9 mL of 0.03 M aqueous sodium acetate, pH 5.2, and then the samples were de-sulfated (Thies 1980) overnight in the column with 100 µL of sulfatase (Sigma). Rinsing with 60 % aqueous methanol eluted the uncharged desulfo-GSs, while unwanted charged compounds remained bonded to Sephadex. Methanol was evaporated from the eluted samples, and then samples were brought to 1 mL with HPLC-grade water. We quantified the GSs in our samples on a Shimazu HPLC system with a LiChrospher (RP-C18, endcapped) 250 × 4 mm analytical column. Injection volume was 20 μL and the flow rate was 1 mL min^−1^. Glucosinolates were eluted using a water–acetonitrile gradient programme with a cleanup and equilibration step at the end. Chromatograms generated at 229 nm were quantified by integrating peak areas. Concentrations were calculated as PA(FW/ITS)/SW, where PA is the sample peak area, FW is the formula weight of the internal standard, ITS is the peak area of the internal standard and SW is the freeze-dried sample weight.

### Offspring generation

#### Experimental design

Two main growth chamber experiments, totalling over 800 plants, were conducted with the offspring of the parental drought treatment groups (CC—control water during rosette and reproductive stages, DC—drought only during the rosette stage and DD—drought treatments during both stages; see Fig. 1 for a description of parent drought treatment groups). The main difference between the two experiments was the arrangement of parental drought treatment groups across flats. In the first experiment, the parental drought treatment groups (CC, DC and DD) varied among flats (i.e. one type of parental drought treatment group per flat). This allowed us to include as many sib-families per parental drought treatment group as possible (*n* = 32) in each flat for more statistical power to detect a genetic tradeoff.

The design of the second offspring experiment reduced the potential confounding effect of the flat for comparison of parental drought treatment groups because offspring of all three parental drought treatment groups were represented within each flat. To do this, we reduced the number of sib-families to 10 and, therefore, focused on the trans-generational plasticity while still controlling for genetic variation (i.e. variation among sib-families). All 10 sib-families and all three parental drought-treatment groups (CC, DC and DD) were represented within each flat.

In the offspring generation, a sib-family represented seed from one sib-family in the parent generation experiment. Therefore, there were no nested effects between generations that needed to be included in statistical analyses. In both offspring experiments, the sib-families used were descendants (grand-offspring) of plants from each of the five field sites and, therefore, were representative of the genetic variation among populations.

#### Offspring drought treatments

Drought treatments (Control and Drought) in both experiments varied among flats as described above in the original experiment with the parent generation. However, in the second experiment with offspring, the drought treatments were prolonged—carried out 45 days longer than the first experiment—in an attempt to intensify the effects of drought.

#### Response variables

Between the two offspring experiments, levels of drought tolerance and defence were assessed in several ways. Experiments were monitored for plant size and growth, and then at the end of the experiments, root and shoot mass, different measures of water-use efficiency (WUE) and shoot GS levels were recorded. The root-to-shoot ratio and WUE are different components or different measures of drought stress tolerance. Water-use efficiency is the ratio of CO_2_ uptake to water loss, but a carbon isotope ratio (δ^13^C) is also used to estimate WUE in C_3_ plants (Farquhar and Richards 1984; McKay *et al*. 2003 and references therein). This is because the ^13^C/^12^C ratio can be modelled as a function of the ratio of intercellular to atmospheric partial pressure of CO_2_ (C_i_/C_a_), which is also supported empirically, and C_i_/C_a_ is empirically correlated with WUE in C_3_ plants. Values of δ^13^C are usually negative, and less negative values mean greater WUE. For carbon isotope discrimination, whole basal rosette shoots were freeze dried, ground to <0.5 mm and analysed with a VG SIRA series II triple trap isotope ratio mass spectrometer (GV Instruments) and values were expressed as per millilitre (‰) ^13^C values. Another measure of WUE is the ratio of accumulated dry biomass to overall water use (Larcher 2003). Therefore, we determined the shoot biomass and water content and used their ratio as a surrogate of another measure of WUE. The water content was determined at the end of the experiment by weighing shoots before and after freeze-drying.

#### Statistical analysis

Both experiments with the offspring were also split-plot in design. To simplify the analyses in the first experiment, we created one whole-flat factor, called D&P, which included all combinations of the three parental drought treatments (CC, DC and DD) and the two offspring drought treatments (Control, Drought). Therefore, the statistical model was
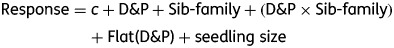

where Response is the particular response variable (e.g. shoot size); D&P is the fixed effect representing the different combinations of offspring and parental drought treatments; Sib-family is a random effect; Flat(D&P) is flat nested within the whole-flat factor of D&P; and seedling size controlled for developmental differences. Again, as in the analysis of the parent generation experiment, the interaction between drought and flat was dropped; therefore, the *F*-ratio for D&P was calculated using MSE_Flat(D&P)_ in the denominator, while all other *F*-ratios were calculated over the MSE_error_.

The statistical model for the second offspring experiment was
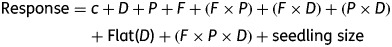

where *D* is the offspring drought treatment (Control or Drought); *P* is the parental drought treatment (CC, DC or DD); *F* is the sib-family and Flat(D) is the flat nested within Drought. Again, the interaction between the drought and the flat was dropped; therefore, the *F*-ratio for Drought was calculated over MSE_Flat(*D*)_, while all other *F*-ratios were calculated over the MSE_error_.

#### Genetic tradeoff detection

To determine whether there was a drought-induced trans-generational effect on the negative genetic correlation between GS allocation and drought tolerance, which we had detected in the parent generation (see Results), in the first offspring generation experiment, we conducted a similar regression analysis on the means of sib-families as described above under ‘Genetic tradeoff detection’. However, in the analysis we added the parental drought treatments and the interaction of GS and parental drought treatment terms to the model.

## Results

### Parent generation

#### Fitness effects of drought

The effect of parental drought treatments on reproduction in the parent generation was dependent on population (Population-by-Drought interactions in the ANCOVAs, Table 1); however, the direct effect of drought was still informative. On average, the drought treatment reduced the basal rosette size by ∼40 %, and in the DDs, delayed flowering by ∼10 % and reduced the reproductive output by ∼30 %. But there were no effects of drought on seed size and thus correlated seed mass (regression of seed mass and size on the sub-sample: *F*_1, 10__=_ 18.154, *P* = 0.002, *r*^2^ = 61 %).

**Table 1.** Significant effects of population (collection site) and drought on the shoot size, flowering time, reproductive output and seed size in the parent generation. *F*-ratios from ANCOVAs are shown. *P ≤ 0.05; **P ≤ 0.01; ***P ≤ 0.001 (significant effects in all ANCOVA tables are indicated in bold).

Source	df	Shoot size	df	Flowering time	Reproductive output	Seed size
Population	5	14.183***	5	33.651***	1.372	21.790***
Drought	1, 10	3.074	2, 9	2.372	1.577	1.003
Pop × Drought	5	2.412*	10	6.637***	2.246*	0.713
Flat(Drought)	10	25.748***	9	4.352***	3.121**	1.334
Seedling size	1	0.253	1	0.088	0.055	
Flowering time			1		57.384***	
Error	306		260			
*r*^2^ (%)		**60.7**		**50.9**	**69.1**	**41.4**

Shoot size was positively correlated with reproductive output across the drought treatments in the parent generation (regression: *F*_1, 308_ = 37.073, *P* < 0.001, *r*^2^ = 22.7 %), and we know from previous work (Siemens *et al*. 2009) that shoot size is also correlated with over-winter survivorship across the range boundary in the field; therefore, plant size in this system can be used as an indicator of fitness and for evolutionary inferences. Reproduction is actually less important across the range where plants of *B. stricta* do not normally occur, and do not usually survive to reproduce in common garden experiments (Siemens *et al*. 2009, 2012). Nonetheless, we were not able to measure reproduction in the offspring generation in the laboratory because tissues were used for GS, carbon isotope ratio and DNA methylation analyses (Methylation results: J. Alsdurf and D. H. Siemens, unpubl. data). Instead, we relied on the above correlations of size and fitness (both reproduction and survivorship) for evolutionary inferences. However, there may be stress-induced trans-generational effects on these correlations between size and fitness, which might limit the use of size as a proxy for fitness. The cost of the trans-generational plasticity might, for example, affect the relationship, but the existence of this cost was not investigated here.

#### Genetic tradeoff

**Figure 2.**
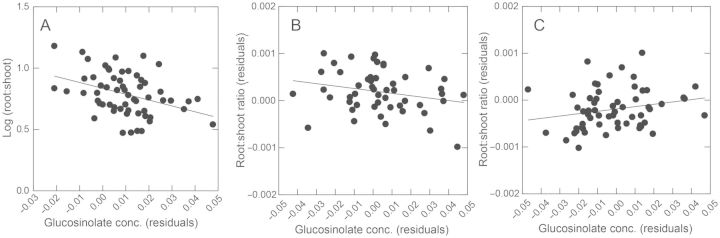
Genetic correlation between the root-to-shoot ratio and 2-hydroxy-1-methylethyl GS concentration in the parent generation (A), and in the offspring whose parents were control watered (B) or drought (DCs shown) treated (C). The trend in the parents was also observed within each population (see Fig. 3A). Data are means of sib-families, controlling as needed for unmeasured random effects among planting flats and for plant size, hence the residuals. In the offspring (B and C) sib-families from both offspring watering treatments appear in each graph.

**Figure 3.**
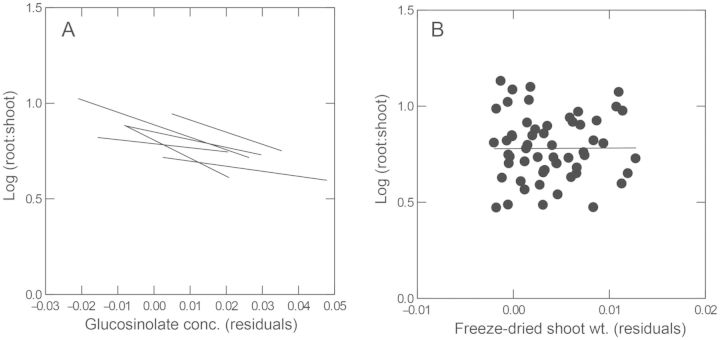
The genetic tradeoff between drought stress tolerance (measured as the root-to-shoot ratio) and GS allocation in the parent generation (Fig. 1A) was observed within each population (A—least squares best fit line for each population is shown). The genetic correlation was not caused by shoot weight, which is used in the calculation of both variables (root-to-shoot ratio and GS concentration), because there was no genetic correlation between the root-to-shoot ratio and the weight of shoot tissue used for GS analysis (B—*F*_1, 57_ = 0.708, *P* = 0.403).

There was a negative genetic correlation between allocation to the most common and abundant 2-hydroxy-1-methylethyl GS and drought tolerance measured as the root-to-shoot ratio (regression analysis on the means of sib-families: *F*_1, 57_ = 9.667, *P* = 0.003, *r*^2^ = 14.5 %, Fig. 2A). The tradeoff was also evident when tolerance was measured as the slope of reaction norms for shoot size across drought treatments (*F*_1, 55_ = 4.463, *P* = 0.039, *r*^2^ = 7.5 %, not shown). Furthermore, the tradeoff was evident among and within all populations, and we controlled for plant size in these analyses (Fig. 3); therefore, the tradeoff is not simply an artefact of GS concentration increasing as plant size decreases (Koricheva 1999). The trends also held for the total GS concentration because 2-hydroxy-1-methylethyl GS and total GS concentrations are highly genetically correlated (*r* = 0.98). However, the trends did not hold (*P* > 0.05) for 1-methylethyl GS, another common, but much less abundant, GS.

### Offspring generation

#### Genetic tradeoff

There was a marginally significant trans-generational effect of parent drought treatments on the genetic correlation (tradeoff) between 2-hydroxy-1-methylethyl GS allocation and root-to-shoot ratio in the offspring generation (regression on the means of sib-families: parent drought treatment-by-GS allocation interaction, *F*_2, 156_ = 3.003, *P* = 0.053, *r*^2^ = 16.5 %). In the offspring, the tradeoff that was apparent in the CC (parental control) group (Fig. 2B) was not apparent in either DC or DD (parental drought-treated) groups (Fig. 2C). Analyses that included offspring drought treatments and their interaction terms were not significant (*P* values > 0.05) and were dropped. Although the patterns were the same for drought tolerance measured as a reaction norm, the effects were not significant (e.g. *F*_2, 61_ = 0.537, *P* = 0.587, *r*^2^ = 5.8 %).

#### Environmental tradeoff

**Figure 4.**
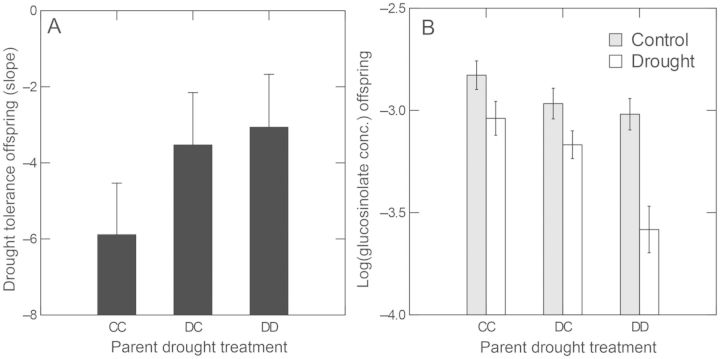
Drought-induced trans-generational effects on tolerance to drought (A). Tolerance was calculated from the difference in shoot size measures between drought and control treatments for each sib-family within each parent treatment group, but drought tolerance was also correlated with the shoot size measured under drought conditions (*F*_1, 72__=_ 81.935, *P* < 0.001). Effects of drought and drought-induced epigenetic effects on the concentration of 2-hydroxy-1-methylethyl GS, the most common GS (B). Error bars indicate standard errors among sib-families or among populations in the case of GSs. Data from statistical analyses are given in Table 2.

Parent drought treatments induced an environmental tradeoff in the offspring generation (Fig. 4). Whereas drought tolerance increased (Fig. 4A), GS levels decreased (Fig. 4B) in the parental drought-treated groups (DC and DD) compared with parental CC controls. This result occurred in both offspring experiments (Fig. 4 shows the result of the first offspring experiment). Furthermore, these effects were clear despite genetic variation in the effects, i.e. significant interactions with sib-family in the ANCOVAs (Table 2 for the first offspring experiment and Table 3 for the second offspring experiment). For example, genetic variation in the drought-induced trans-generational effect on drought tolerance was indicated by the three-way interaction between sib-family and parental and offspring drought treatments in the analysis for shoot size (Table 2).

**Table 2.** Significant effects of drought treatments (Parental-P and Offspring-D) and sib-family (genetic variation) on shoot size, root dry weight, root-to-shoot ratio and GS concentration in the first offspring experiment. *F*-ratios from ANCOVAs are shown. *P ≤ 0.05; **P ≤ 0.01; ***P ≤ 0.001. ^a^D&P term represents all combinations of offspring drought- (control and drought) and parent drought (CC, DC and DD) treatments. ^b^The results for 2-hydroxy-1-methylethyl, the most common and abundant GS. Analysis for 1-methylethyl GS, the second most common GS, was similar (see the text). ^c^In the case of GS concentration, population was used instead of sib-family in the analysis because effects of sib-family were not readily detected.

Source	df	Shoot size	Root dry weight	Root-to-shoot ratio	df	GS^b^ concentration
D&P^a^	5, 6	4.903*	0.465	0.273	5, 6	0.942
Sib-family^c^	25	7.575***	2.093**	2.331**	4^c^	4.319**
D&P × Sib-fam	125	1.366*	1.021	1.332*	20^c^	1.787*
Flat(D&P)	6	3.204**	20.624***	21.006***	6	7.741***
Seedling size	1	1.206	0.830	0.028	1	1.426
Error	143				189	
*r*^2^ (%)		77.4	73.1	74.2		40

**Table 3.** Significant effects of offspring drought treatments (D), parental drought treatments (P) and sib-family (Fam) on the growth of shoots, shoot dry weight, shoot mass per water content, carbon isotope ratio (δ^13^C) and GS concentration in the second offspring experiment. *F*-ratios from ANCOVAs are shown. *P ≤ 0.05; **P ≤ 0.01; ***P ≤ 0.001. ^a^Error df for shoot dry weight and specific water content were each 173 because of drought mortality, and 80 for Δ^13^C because we reduced the number of controls to match the number of survivors. ^b^Insufficient sample size to include the three-way interaction for Δ^13^C analysis. ^c^*df* for MANCOVA (multivariate analysis of co-variance) conducted on the three most common glucosinolates (GS). Sib-family was not included in the analysis.

Source	df	Growth rate	Shoot dry weight	Mass/H_2_O	δ^13^C	df^c^	GS
D	1, 12	78.450***	5.690*	0.216	12.553**	3, 10	1.307
P	2	0.096	0.364	0.038	1.700	6, 280	3.214**
Fam	6	4.659***	2.160*	2.110*	8.931***		
Fam × P	12	3.133**	1.311	1.257	2.692**		
Flat(D)	12	3.599***	3.414***	3.184***	2.423**	36, 414	4.575***
D × P	2	0.860	0.040	0.049	0.935	6, 280	3.889***
Fam × D	5	3.133**	0.889	1.888	1.459		
Fam × D × P	11	1.775*	1.243	1.926*	^b^		
Seedling size	1	0.001	0.000	0.750	0.000	3, 140	0.298
Final dry mass	1			1.231			
Error	265^a^						
*r*^2^ (%)		62.2	68.2	40.8	69.5

#### Drought tolerance traits

**Figure 5.**
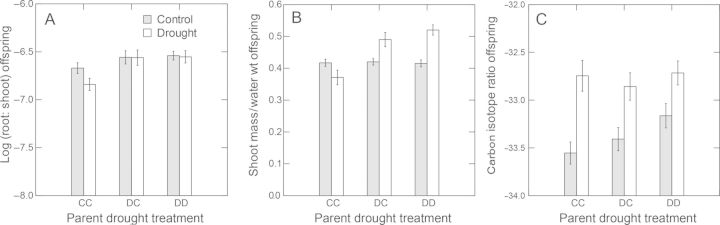
Effects of parental and offspring drought treatments on root-to-shoot ratios (A), shoot biomass per water content (B) and carbon isotope ratios (C). Grey bars in all three graphs represent control offspring watering treatments and open bars represent drought treatments. For root-to-shoot ratios, data on statistical analyses are given in Table 2, otherwise see Table 3. Log (root:shoot) are residuals after controlling for flat and development. Error bars are standard errors among sib-family means.

The other measures or components of drought tolerance in the offspring generation, root-to-shoot ratio and WUE, were similarly affected by parental drought treatments (Fig. 5). Offspring whose parents had been drought treated had higher root-to-shoot ratios even under control watering conditions (Fig. 5A). Again there was significant genetic variation in the trans-generational effects of drought (three-way interaction with the sib-family in the ANCOVA, Table 2).

Water-use efficiency measured as accumulated dry shoot biomass per water content at the end of the experiment increased under drought conditions in the offspring whose parents had been drought treated, while the opposite was true for the offspring whose parents had been control treated (Fig. 5B). Again, there was genetic variation in the trends (three-way interaction among family, offspring drought and parental drought treatments, Table 3). This measure of WUE was phenotypically correlated with WUE measured as carbon isotope ratio (*δ*^13^C) (regression: *F*_1, 98_ = 16.901, *P* < 0.001, *r*^2^ = 14.7 %, mass/H_2_O = 0.032 + 0.384 δ^13^C).

Water-use efficiencies measured as carbon isotope ratio were high among all of the parent drought-treatment groups under drought conditions (*P* < 0.01, Table 3), indicating drought avoidance through closed stomata, while under control watering conditions, carbon isotope ratios seemed to be higher in the offspring whose parents had been drought treated (Fig. 5C); however, sample sizes were insufficient to include the three-way interaction among drought, parental drought-treatment group and family that probably would have been required to detect significance (Table 3). Sample sizes were lower because the shoot tissue was used in other analyses (e.g. GSs).

The prolonged and strong drought treatment in the second offspring experiment eventually resulted in 61 % mortality among the drought-treated plants. Although we did not detect any significant differences among the parental drought-treatment groups in offspring survivorship (logistic regression: *Z* = 1.304, *P* = 0.192), we did detect differences among the survivors in terms of performance. There were more plants among the survivors in the DC and DD groups that were larger and greener (blind subjective census, and logistic regression: *Z* = 2.845, *P* = 0.004). Plants that were less green had turned reddish purple, probably from drought-induced anthocyanin accumulation (Chalker-Scott 1999).

## Discussion

The adaptive significance of trans-generational plasticity in range limits remains largely unexplored (see Sexton *et al*. 2009 for review on range limits). Here, we examined the role of stress-induced trans-generational plasticity in mitigating a putative genetic constraint that may contribute to low-elevation range limits in *B. stricta* (Siemens *et al*. 2009). Although we found some evidence that stress-induced trans-generational effects may overcome a genetic constraint (Fig. 2), we suggest that the ‘environmental tradeoff’ detected (Fig. 4) may actually contribute to the prevention of successful colonization outside the normal range. Whereas the drought-induced trans-generational increase in drought tolerance (Fig. 4A) would likely be adaptive in the drier areas across the range boundary, the decrease in GS (Fig. 4B) would likely be maladaptive, hence the tradeoff.

In another study that has examined the role of trans-generational plasticity in the use of marginal habitats, Stanton and Galen (1997) found that early snow melt, and thus a longer growing season, among other factors, increased seed provisioning, which enabled offspring of the snow buttercup (*Ranunculus adoneus*) to better survive adjacent, occupied, marginal habitats. They argued that this created gene flow that prevented adaptation to the marginal sites where snow melted off late. In contrast, we did not find that the benign control watering treatments of parents benefited offspring under drought conditions. Instead, we found the opposite to be true, that is, the drought stress treatments of the parents benefited offspring under drought conditions.

The effects of trans-generational induction are often similar, at least superficially, to plastic stress responses of an individual plant within a generation. For example, for induced defence responses against pests, there are now several examples of trans-generational induction of resistance to herbivores or pathogens (see Holeski *et al*. 2012 for review). When an individual plant of *B. stricta* was drought stressed, we found that GS levels remained the same or decreased, mainly depending on the genetic variation. Of the relatively few studies that have investigated the effects of drought on GS levels within a generation, most have also found GS levels to decrease (e.g. Khan *et al*. 2010; Gutbrodt *et al*. 2011; Mewis *et al*. 2012), but the results vary with the type of GS and species or cultivar (e.g. Schreiner *et al*. 2009). In one case, induction of GS in a cultivar of *Brassica oleracea* by a generalist aphid was also attenuated under drought conditions (Khan *et al*. 2011). Thus, within a generation, it appears that a drought stress tolerance response in mustards may coincide with a reduction in some defences, including induced defence responses. But this generality is complicated by the function that some GS may have in osmoregulation (Schreiner *et al*. 2009).

### Evolutionary implications

We have thus far used the term adaptive in evaluating the offspring and their traits, not the trans-generational plasticity *per se* (see below). To justify this use, the traits must be correlated with fitness. Drought tolerance measured as the root-to-shoot ratio or a shoot size-based reaction norm was correlated with reproductive output in the laboratory (see above under Results, Parent generation), survivorship across the range boundary in the field (Siemens *et al*. 2009), and was even manifest under control watering conditions (e.g. Fig. 5A). In another study, we have also found parental drought treatments to increase offspring reproduction (number of fruits per plant) by 10.1 % even under control watering conditions (D. H. Siemens, T. J. Ripley and J. D. Alsdurf, unpublished growth chamber experiment: *F*_1, 97_ = 7.127, *P* = 0.009). Thus, the higher drought tolerance would likely be adaptive in the drier environments across the range boundaries at lower elevations, although field tests are needed.

Similarly, the drought-induced trans-generational decrease in GS would likely be maladaptive across the range boundary because, in transplant experiments, we have found that attack by generalist insect herbivores is more frequent in drier environments across the low-elevation range boundaries, and that plants that have inherently high levels of certain GS are attacked less (Siemens *et al*. 2009). Furthermore, even minimal experimental or natural herbivore damage to *B. stricta* can result in lower fitness in the field, but GSs reduce the damage by generalists (Prasad *et al*. 2012).

As in other studies on trans-generational induction (see Herman and Sultan 2011 for review), there was genetic variation for all of the trans-generational induced effects observed, suggesting that there is evolutionary potential for the trans-generational plasticity *per se*. Because the low-elevation *B. stricta* populations in this study may have already evolved to cope some with relatively dry environments, one might have predicted that there would be adaptive trans-generational drought-induced responses for drought-tolerant traits, as we observed. Stress-induced trans-generational plasticity is adaptive if parental stress changes the development of offspring traits to improve growth and fitness in response to the same stressor (Herman and Sultan 2011). Furthermore, adaptive trans-generational plasticity may evolve when the parent environment is a reliable predictor of the offspring environment. For example, Sultan *et al*. (2009) experimentally drought-treated parents and found that offspring of the weed *Polygonum persicaria*, which naturally occurs in heterogeneous habitats that include drier sites, had greater root growth and higher biomass under water-deficient conditions than did *P. hydropiper*, a closely related congener that is restricted naturally to more moist conditions.

## Conclusions

In the study of range limit development, the focus has been on leading edge instead of trailing edge dynamics (Sexton *et al*. 2009). This is an important caveat as trailing edge dynamics will be critical for understanding population-level persistence. Our study begins to fill this knowledge gap and extends the conceptual framework of the field by focusing on trans-generational environmental effects. We found that while these effects may overcome some constraints on stress tolerance evolution and range expansion, other constraints may be created to limit range. This adaptive–maladaptive duality of the tans-generational environmental effects involved the same two traits, defence and stress tolerance. If our specific hypothesis is correct, that defence allocation contributes to range limit development by negatively affecting stress tolerance, this would help explain why mustards (Brassicaceae), which all contain GSs, generally inhabit high-altitude temperate regions where populations have patchy distributions (I. A. Al-Shehbaz, pers. commun.). Such distributions would lend themselves to isolated populations and thus genetic divergence, which might also help explain the high diversity of mustard species.

## Sources of Funding

Our work was supported in part by an Institutional Development Award (IDeA) from the National Institute of General Medical Sciences of the National Institutes of Health under grant number P20GM103443. The content is solely the responsibility of the authors and does not necessarily represent the views of NIH.

## Contributions by the Authors

Experiments were carried out by J.D.A., T.J.R. and D.H.S., glucosinolate extraction and quantification were carried out by T.J.R. and D.H.S. and carbon isotope ratios were determined by S.L.M. Statistical analyses were conducted by J.D.A. and D.H.S., J.D.A. and D.H.S. wrote the manuscript, with insightful comments by all co-authors.

## Conflict of Interest Statement

None declared.
